# Swivel and Roll! - A new dance for the modern Thoracic Surgeon

**DOI:** 10.1186/1749-8090-10-S1-A203

**Published:** 2015-12-16

**Authors:** Sparsh Prasher, Michael Klimatsidas

**Affiliations:** 1 Golden Jubilee National Hospital, Agamemnon St, Clydebank, Dunbartonshire G81 4DY, UK

## Background/Introduction

In comparison to open surgery, VATS lung resections are technically challenging operations as the operative field is transformed from a three dimensional multi-angle wide view to a two dimensional screen. Therefore careful pre-operative and intra-operative planning is required to complete the procedure safely and successfully. All our patients undergo a Computed Tomography (CT) which is essential for pre-operative planning.

The Multiplanar Reconstruction (MPR) mode displays images in the Coronal and Sagittal view. The sagittal view can be rotated by 90 degrees to bring the lung apex to the left side. This corresponds to the patient's position in the right lateral decubitus position (for left sided resections) but not in the left lateral decubitus position for right sided resections. Therefore, the standard sagittal view in the MPR mode is not adequate for operative planning for right sided lung resections.

## Aims/Objectives

To improve pre-operative planning for VATS lung resections by introducing a new way of manipulating the CT images.

## Method

We used the Swivel function to manipulate the images in the coronal axis to produce a sagittal view so that the spine lies on the left hand side and the sternum on the right side. The roll function allows the image in the sagittal view to move by 90 degrees this orientates the images in the patient's position on the operating table.

## Results

The results are demonstrated in the images below.

**Figure 1:**
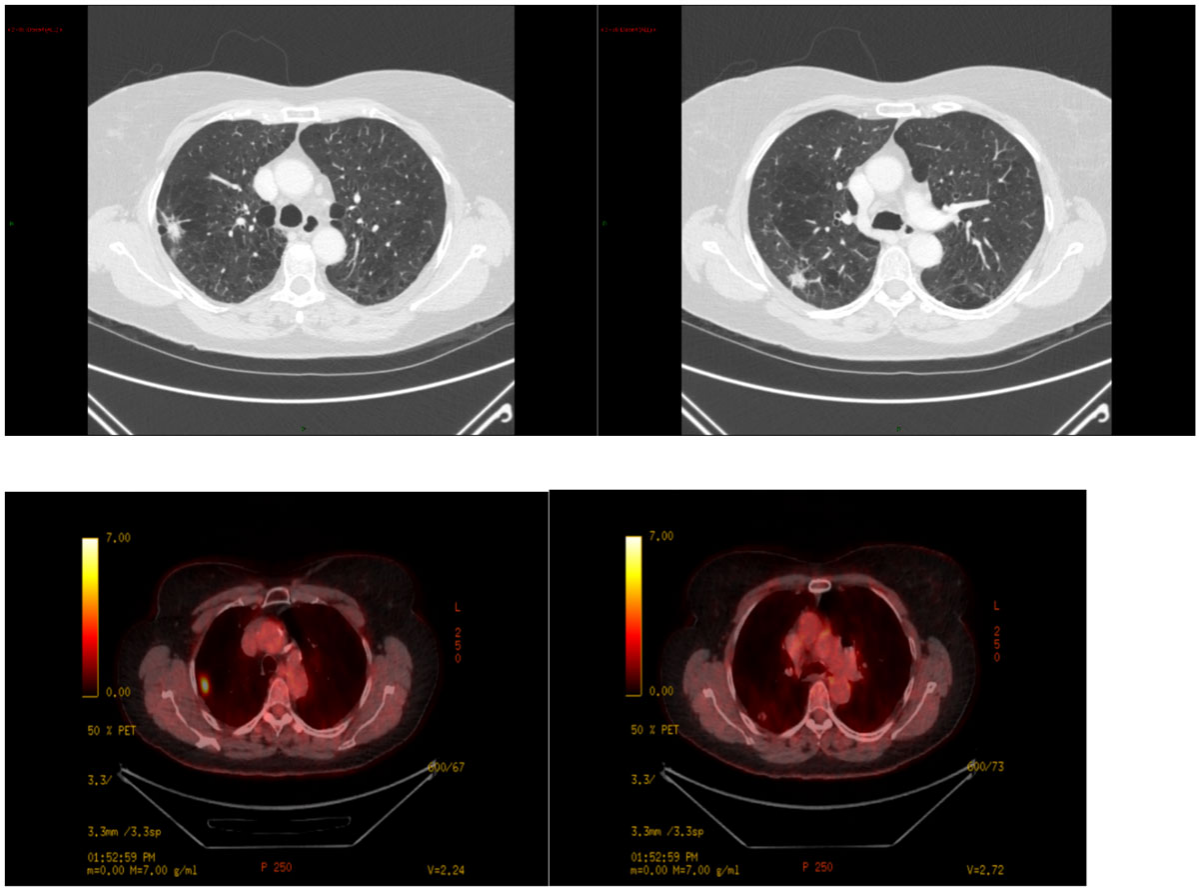
**Two Right sided lung lesions**. But only one is FDG Avid on the PET scan.

**Figure 2:**
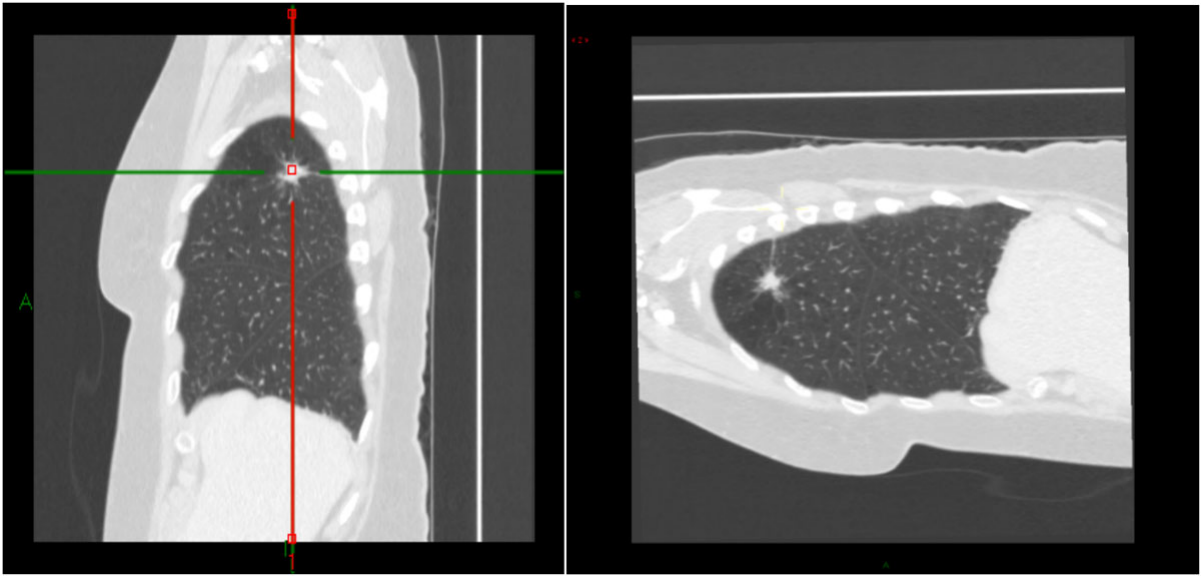
**Pre Swivel orientation in MPR mode**. Note that in the sagittal view the Spine is on the right side and sternum on the left. The Right upper lobe is on the left of the screen but on the right of the surgeon.

